# Gastrointestinal Amyloidosis: Review of the Literature

**DOI:** 10.7759/cureus.1228

**Published:** 2017-05-08

**Authors:** Kyle Rowe, Jon Pankow, Fredy Nehme, William Salyers

**Affiliations:** 1 Internal Medicine, University of Kansas School of Medicine - Wichita

**Keywords:** gastrointestinal amyloidosis, amyloidosis

## Abstract

Gastrointestinal amyloidosis (GIA), a protein deposition disorder, represents a complex common pathway that encompasses multiple etiologies and presentations. It represents a significant diagnostic and treatment challenge. The disease results from the deposition of insoluble extracellular protein fragments that have been rendered resistant to digestion. GIA can be acquired or genetic, and most commonly results from chronic inflammatory disorders (AA amyloidosis), hematologic malignancy (AL amyloidosis), and end-stage renal disease (Beta-2 amyloidosis). The deposition of these abnormal proteins interferes with gastrointestinal tract (GI) organ structure and function, most notably in the liver and small bowel. Presentation from GI involvement includes cirrhotic sequelae, abdominal pain, malabsorption, and GI bleeding. Diagnosis hinges on pathologic examination of affected tissue, with classic green birefringence under polarized light. Abdominal fat pad and rectal mucosal biopsy have been described as sites of higher sensitivity for diagnosis. Serum amyloid P scintigraphy is near 90% sensitive for diagnosis of AA amyloidosis. Patients should be considered for further evaluation to rule out additional organ involvement, notably cardiac and renal. Treatment hinges on an adequate suppression of the predisposing inflammatory disorder, or malignancy, followed by supportive therapy. Prognosis varies depending on the etiology of the disease, with the AL subtype showing worse outcomes, as well as those with hepatic involvement.

## Introduction and background

Gastrointestinal amyloidosis, a protein deposition disorder is a complex and diverse entity with multiple etiologies and presentations. It represents a significant diagnostic and treatment challenge. In this review, we aim to describe the current understanding of pathophysiology, epidemiology, clinical presentation, diagnosis, workup, treatment, and prognosis of gastrointestinal amyloidosis.

## Review

### Pathophysiology

The term amyloidosis is used to describe a pathologic finding, which in fact encompasses a heterogeneous spectrum of etiologies and clinical presentations. The hallmark of amyloidosis is the deposition of insoluble extracellular protein fragments within various organs, abnormally folded in such a way that renders them resistant to digestion [1]. This deposition impairs both the structure and function of the affected organs. This process can be either genetic or acquired, though this review will focus only on the acquired forms. While many proteins have the potential to form amyloid fibrils, the most commonly acquired amyloid precursors are immunoglobulin derived light chain (AL amyloidosis), serum amyloid A (AA amyloidosis, an acute phase reactant), b-2 microglobulin (Ab2m, dialysis-related), and apolipoprotein E (Alzheimer’s related) [2-3]. Acquired amyloidosis results most commonly from a hyperproteinemic state originating from either plasma cell dyscrasia (AL) or a chronic inflammatory state (AA). Serum amyloid A is an acute phase reactant, present in excess in these states. In the case of dialysis-related amyloidosis, protein accumulation is due to decrease renal clearance of b-2 microglobulin [4]. Each type of amyloidosis shows varying predilections to deposit in target organs, with AA amyloidosis showing the highest rates of gastrointestinal (GI) manifestations at 10%-70%. AL amyloidosis, for unclear reasons, has been reported to cause fewer extrahepatic GI manifestations [5].

Within the GI tract, amyloid deposition occurs in the muscularis mucosae, within close proximity to vasculature, nerves, and nerve plexuses [6]. This deposition increases the frailty of blood vessels, hinders intrinsic peristalsis and decreases the compliance of the gut wall [7]. These mechanisms combined are likely responsible for the symptomatology seen in GI amyloidosis. In the liver, amyloid deposition within hepatic stellate cells results in activation and promotion of a fibrogenic state which can be measured by Elastography [8]. The resulting mechanical and functional sequelae are comparable to other fibrotic liver diseases.

### Epidemiology

The epidemiology of amyloidosis is incompletely described due to its rare prevalence in addition to the wide spectrum of etiologies and manifestations. Additionally, regional variations in genetic and environmental contributors affect the likelihood of developing the disease. Such variations include polymorphisms within genes encoding amyloid precursors, as well as the prevalence of local infectious and autoimmune disease which predispose to chronic inflammation. This may confound any estimates regarding incidence.

One epidemiologic study in Minnesota reviewed a 40-year interval and reported the overall prevalence of AL amyloidosis to be between six and 10 cases per million person-years, correlating to approximately 2,200 annual cases in the United States [2]. A Latin American study reported the incidence densities in Buenos Aires to be 6.13 (95%CI: 2.57-9.7) per million person-years for AL and 1.21 per million person-years (95%CI: 0.56 to 2.99) for AA amyloidosis [9]. Similar results have been found in recent European studies [10-11].

Acquired amyloidosis most commonly manifests later in life, between 45-64 years of age, and the age of onset appears to be increasing for AA amyloidosis as treatments for predisposing inflammatory and infectious states to improve [3].

### Clinical presentation

The presentations of systemic amyloidosis are variable and dependent on the degree of organ involvement. Cardiac, peripheral neurologic, and renal manifestations are highly prevalent [12].

Several longitudinal and cross-sectional cohorts have described the symptomatology of GI amyloidosis. Common presenting symptoms include weight loss, diarrhea, abdominal pain, malabsorption, esophageal reflux, and varying degrees of upper and lower GI bleeding, including fatal hemorrhage [13-16]. Hepatic symptoms include jaundice, steatorrhea, anorexia, and those related to portal hypertension such as ascites and splenomegaly [17-18].

### Diagnosis and workup

The gold standard for diagnosing amyloidosis is tissue biopsy of an affected organ with congo red stain demonstrating green birefringence under polarized light [19]. Given the non-specific presentation, many patients will undergo extensive testing prior to biopsy. Attention must be given to family history and knowledge of conditions that predispose to amyloid formation. A hepatic biopsy is discouraged if tissue can be obtained elsewhere due to the risk of hemorrhage. Abdominal fat pat aspirate has been performed, though studies demonstrate highly variable sensitivity, from 13% to 73%, with higher sensitivities noted in the AL and genetic subtypes [20-21]. Specificity in these studies was 100%. In those requiring endoscopy, a rectal mucosal biopsy is a reasonable screening option with the sensitivity of 75%-85% [22]. Biopsy specimens should undergo further testing to determine amyloid subtype to further guide management [23]. Whole-body 123I-labeled serum amyloid P (SAP) scintigraphy is up to 90% sensitive for diagnosis of systemic AA amyloidosis and can reveal the degree of organ involvement, though cardiac involvement cannot be determined by this method [24]. If systemic amyloidosis is suspected or confirmed, patients should undergo screening for bone marrow malignancy, cardiomyopathy, and nephropathy [23, 25].

Those presenting with GI manifestations often have indications for endoscopy such as weight loss, dyspepsia refractory to medical therapy or GI bleeding [26-27]. Any site can be affected, but the small bowel is most commonly affected [28]. Endoscopic appearance may be highly variable and there have been disputing reports of the correlation between the type of amyloidosis and endoscopic findings. One study involving 30 patients found AL amyloidosis to be more associated with bulk deposition, causing mucosal protrusions and thickened intestinal folds, while AA amyloidosis was more associated with diffuse deposition causing mucosal friability and ulcerations [29-30]. A separate study, however, found no correlation between the type of amyloidosis and endoscopic findings [31]. In the stomach, findings can include submucosal tumor-like masses, erosions, ulcerations, intramural hematomas, and rugal thickening [32]. Amyloidomas have been reported on endoscopy or endoscopic ultrasound in the pancreas, stomach, and rectum [33-35].

### Treatment

The cornerstone of treatment for acquired amyloidosis is the treatment of the underlying disorder causing elevated amyloid precursors, whether malignancy, infection or autoimmune disease [36-38]. AL amyloidosis with evidence of organ involvement is an indication for treatment of any monoclonal gammopathy regardless of clone burden [36]. These patients benefit from oncology consultation for consideration of chemotherapy and autologous stem-cell transplant.

There are no identified randomized controlled trials available to guide therapy in AA amyloidosis. Based on retrospective data and theory, agents with optimal activity against the underlying process typically yield the best results, most commonly with biologic therapies [3, 39-40]. There have been numerous reports of inducing successful disease remission using Tocilizumab, a monoclonal antibody directed against IL-6 the receptor [41]. One retrospective study of 42 patients with AA amyloidosis due to various rheumatic diseases found that after five years, 72.7% of those treated with anti-Interleukin 6 receptor (IL-6) agents were in clinical and laboratory remission compared to 40.7% of those on anti-tumour necrosis factor therapy (TNF) [42]. However, several reports have described the good response to anti-TNF therapy, especially in those with inflammatory bowel disease [37, 43]. For those with severe hepatic disease and often in genetic types, the liver transplant can be a viable option [44-45].

For gastrointestinal sequelae, supportive therapy involves nutritional and vitamin supplementation for those with malabsorption, treatment of diarrhea or obstruction, and addressing any resulting infections from treatment-related immune compromise. Octreotide has been used successfully for refractory diarrhea and protein-losing enteropathy [46-48]. Surgical resection may be required in cases of large GI hemorrhage or obstruction [49].

### Prognosis

Prognosis depends on the underlying etiology for amyloid deposition, as well as the degree of organ involvement. AL amyloidosis typically is associated with a worse prognosis due to its association with underlying malignancy. One prospective cohort of 137 patients with AL amyloidosis found median survival times of 15.84 months for those without GI involvement, and 7.95 months for those with GI involvement [50]. An additional cohort of 374 patients with AA amyloidosis showed a median survival of 133 months (95%CI 100-153) [12]. The presence of hepatic amyloid deposits in this cohort portended adjusted relative risk of death of 1.98 [12]. In those with AA amyloidosis where effective treatment hinges on addressing the underlying inflammatory or infectious disorder, tracking the deposition of amyloid using whole-body 123I-labeled serum amyloid P component (SAP) scintigraphy could prove useful. In the previous cohort, the demonstration of regressing deposits via this method was associated with a 0.15 relative risk (RR) of mortality [12].

## Conclusions

Systemic amyloidosis encompasses a wide variety of etiologies and manifestations. Within the GI tract, symptoms are often non-specific and range from nausea, reflux, and diarrhea, to more severe hemorrhage and obstruction. Within the liver, amyloidosis represents a fibrotic disease with associated manifestations. Clinicians should maintain a high suspicion in patients predisposed, namely hematologic malignancy, chronic infection, and autoimmune disease, however, symptoms of amyloidosis may in some cases be the initial manifestation of the underlying process. Diagnosis depends largely on tissue biopsy and endoscopy and is often indicated for those with GI symptoms. Workup should include ruling out other organ involvement, notably cardiac and renal. Treatment is based on addressing the underlying etiology for elevated amyloid precursors and most often involves chemotherapy or biologic therapy. Prognosis depends on subtype. AL subtype typically shows worse prognosis than AA subtype, and those with GI involvement typically have worse outcomes. 

## References

[1] Sipe JD, Benson MD, Buxbaum JN (2014). Nomenclature 2014: Amyloid fibril proteins and clinical classification of the amyloidosis. Amyloid.

[2] Kyle RA, Linos A, Beard CM (1992). Incidence and natural history of primary systemic amyloidosis in Olmsted County, Minnesota, 1950 through 1989. Blood.

[3] Real de Asua D, Costa R, Galvan JM (2014). Systemic AA amyloidosis: epidemiology, diagnosis, and management. Clin Epidemiol.

[4] Jadoul M, Drueke TB (2016). Beta2 microglobulin amyloidosis: an update 30 years later. Nephrol Dial Transplant.

[5] Petre S, Shah IA, Gilani N (2008). Review article: gastrointestinal amyloidosis - clinical features, diagnosis and therapy. Aliment Pharmacol Ther.

[6] Kaiserling E, Krober S (1995). Massive intestinal hemorrhage associated with intestinal amyloidosis. An investigation of underlying pathologic processes. Gen Diagn Pathol.

[7] Hirschfield GM (2004). Amyloidosis: a clinico-pathophysiological synopsis. Semin Cell Dev Biol.

[8] Matsuda S, Motosugi U, Kato R (2016). Hepatic amyloidosis with an extremely high stiffness value on magnetic resonance elastography. Magn Reson Med Sci.

[9] Aguirre MA, Boietti BR, Nucifora E (2016). Incidence rate of amyloidosis in patients from a medical care program in Buenos Aires, Argentina: a prospective cohort. Amyloid.

[10] Pinney JH, Smith CJ, Taube JB (2013). Systemic amyloidosis in England: an epidemiological study. Br J Haematol.

[11] Hemminki K, Li X, Forsti A (2012). Incidence and survival in non-hereditary amyloidosis in Sweden. BMC Public Health.

[12] Lachmann HJ, Goodman HJ, Gilbertson JA (2007). Natural history and outcome in systemic AA amyloidosis. N Engl J Med.

[13] Menke DM, Kyle RA, Fleming CR (1993). Symptomatic gastric amyloidosis in patients with primary systemic amyloidosis. Mayo Clin Proc.

[14] Fonnesu C, Giovinale M, Verrecchia E (2009). Gastrointestinal amyloidosis: a case of chronic diarrhoea. Eur Rev Med Pharmacol Sci.

[15] Kim SH, Kang EJ, Park JW (2009). Gastrointestinal amyloidosis presenting with multiple episodes of gastrointestinal bleeding. Cardiovasc Intervent Radiol.

[16] Ebert EC, Nagar M (2008). Gastrointestinal manifestations of amyloidosis. Am J Gastroenterol.

[17] Sonthalia N, Jain S, Pawar S (2016). Primary hepatic amyloidosis: A case report and review of literature. World J Hepatol.

[18] Shin YM (2011). Hepatic amyloidosis. Korean J Hepatol.

[19] Katoh N, Matsuda M, Ikeda S (2015). Clinical, endoscopic, and histopathological features of localized immunoglobulin light chain (AL) amyloidosis in the gastrointestinal tract. Amyloid.

[20] Ikeda S, Sekijima Y, Tojo K (2011). Diagnostic value of abdominal wall fat pad biopsy in senile systemic amyloidosis. Amyloid.

[21] Guy CD, Jones CK (2001). Abdominal fat pad aspiration biopsy for tissue confirmation of systemic amyloidosis: specificity, positive predictive value, and diagnostic pitfalls. Diagn Cytopathol.

[22] Hachulla E, Grateau G (2002). Diagnostic tools for amyloidosis. Joint Bone Spine.

[23] Gillmore JD, Wechalekar A, Bird J (2015). Guidelines on the diagnosis and investigation of AL amyloidosis. Br J Haematol.

[24] Hazenberg BP, van Rijswijk MH, Piers DA (2006). Diagnostic performance of 123I-labeled serum amyloid P component scintigraphy in patients with amyloidosis. Am J Med.

[25] Gertz MA, Benson MD, Dyck PJ (2015). Diagnosis, prognosis, and therapy of transthyretin amyloidosis. J Am Coll Cardiol.

[26] Madsen LG, Gimsing P, Schiodt FV (2009). Primary (AL) amyloidosis with gastrointestinal involvement. Scand J Gastroenterol.

[27] Lam PW, Lam YS, Liu HW (2016). Gastrointestinal: A rare cause of bloody diarrhoea - Gastrointestinal amyloidosis. J Gastroenterol Hepatol.

[28] Syed U, Ching Companioni RA, Alkhawam H (2016). Amyloidosis of the gastrointestinal tract and the liver: clinical context, diagnosis and management. Eur J Gastroenterol Hepatol.

[29] Hokama A, Kishimoto K, Nakamoto M (2011). Endoscopic and histopathological features of gastrointestinal amyloidosis. World J Gastrointest Endosc.

[30] Tada S, Iida M, Yao T (1994). Endoscopic features in amyloidosis of the small intestine: clinical and morphologic differences between chemical types of amyloid protein. Gastrointest Endosc.

[31] Alcalde-Vargas A, Leo-Carnerero E, Rojas-Mercedes N (2015). Correlation between location of amyloid deposits and endoscopic and clinical manifestations in symptomatic gastrointestinal amyloidosis. Rev Esp Enferm Dig.

[32] Sawada T, Adachi Y, Akino K (2012). Endoscopic features of primary amyloidosis of the stomach. Endoscopy.

[33] Sparks D, Bhalla A, Dodge J (2014). Isolated gastric amyloidoma in the setting of marginal zone MALT lymphoma: case report and review of the literature. Conn Med.

[34] Jiang H, Pulinthanathu R, Yee-Chang M (2015). Pancreatic amyloidoma associated with elevated CA19-9: A case diagnosed by endoscopic ultrasound-guided fine-needle aspiration biopsy. Diagn Cytopathol.

[35] Sharma R, George VV (2015). Transanal endoscopic microsurgery: the first attempt in treatment of rectal amyloidoma. World J Gastroenterol.

[36] Dispenzieri A, Buadi F, Kumar SK (2015). Treatment of immunoglobulin light chain amyloidosis: mayo stratification of myeloma and risk-adapted therapy (msmart) consensus statement. Mayo Clin Proc.

[37] Tosca Cuquerella J, Bosca-Watts MM, Anton Ausejo R (2016). Amyloidosis in inflammatory bowel disease: a systematic review of epidemiology, clinical features, and treatment. J Crohns Colitis.

[38] Poullos PD, Stollman N (2003). Gastrointestinal Amyloidosis: Approach to Treatment. Curr Treat Options Gastroenterol.

[39] Hamanoue S, Suwabe T, Hoshino J (2016). Successful treatment with humanized anti-interleukin-6 receptor antibody (tocilizumab) in a case of AA amyloidosis complicated by familial Mediterranean fever. Mod Rheumatol.

[40] Keersmaekers T, Claes K, Kuypers DR (2009). Long-term efficacy of infliximab treatment for AA-amyloidosis secondary to chronic inflammatory arthritis. Ann Rheum Dis.

[41] Courties A, Grateau G, Philippe P (2015). AA amyloidosis treated with tocilizumab: case series and updated literature review. Amyloid.

[42] Okuda Y, Ohnishi M, Matoba K (2014). Comparison of the clinical utility of tocilizumab and anti-TNF therapy in AA amyloidosis complicating rheumatic diseases. Mod Rheumatol.

[43] Akdogan MF, Gucun M, Denizli N (2011). Complete reversal of nephrotic syndrome secondary to amyloidosis with use of infliximab in a patient with inflammatory bowel disease and ankylosing spondylitis. Ren Fail.

[44] Elnegouly M, Specht K, Zoller H (2016). Liver transplantation followed by autologous stem cell transplantation for acute liver failure caused by AL amyloidosis. Case report and review of the literature. Ann Hepatol.

[45] Ueno A, Katoh N, Aramaki O (2016). Liver transplantation is a potential treatment option for systemic light chain amyloidosis patients with dominant hepatic involvement: a case report and analytical review of the literature. Intern Med.

[46] Shin JK, Jung YH, Bae MN (2013). Successful treatment of protein-losing enteropathy due to AA amyloidosis with octreotide in a patient with rheumatoid arthritis. Mod Rheumatol.

[47] Fushimi T, Takahashi Y, Kashima Y (2005). Severe protein losing enteropathy with intractable diarrhea due to systemic AA amyloidosis, successfully treated with corticosteroid and octreotide. Amyloid.

[48] Yam LT, Oropilla SB (1991). Octreotide for diarrhea in amyloidosis. Ann Intern Med.

[49] Rives S, Pera M, Rosinol L (2002). Primary systemic amyloidosis presenting as a colonic stricture: successful treatment with left hemicolectomy followed by autologous hematopoietic stem-cell transplantation: report of a case. Dis Colon Rectum.

[50] Lim AY, Lee JH, Jung KS (2015). Clinical features and outcomes of systemic amyloidosis with gastrointestinal involvement: a single-center experience. Korean J Intern Med.

